# Cracking the Breast Cancer Glyco-Code through Glycan-Lectin Interactions: Targeting Immunosuppressive Macrophages

**DOI:** 10.3390/ijms22041972

**Published:** 2021-02-17

**Authors:** Nuno Lopes, Viviana G. Correia, Angelina S. Palma, Catarina Brito

**Affiliations:** 1 iBET, Instituto de Biologia Experimental e Tecnológica, Apartado 12, 2781-901 Oeiras, Portugal; nlopes@ibet.pt; 2 Instituto de Tecnologia Química e Biológica António Xavier, Universidade Nova de Lisboa, Av. da República, 2780-157 Oeiras, Portugal; 3 UCIBIO, Departamento de Química, NOVA School of Science and Technology, FCT-NOVA, 2829-516 Caparica, Portugal; vivianacorreia@campus.fct.unl.pt

**Keywords:** breast cancer, tumour microenvironment, tumour-associated macrophages, aberrant glycosylation, glycan-lectin interactions, immunotherapy

## Abstract

The immune microenvironment of breast cancer (BC) is composed by high macrophage infiltrates, correlated with the most aggressive subtypes. Tumour-associated macrophages (TAM) within the BC microenvironment are key regulators of immune suppression and BC progression. Nevertheless, several key questions regarding TAM polarisation by BC are still not fully understood. Recently, the modulation of the immune microenvironment has been described via the recognition of abnormal glycosylation patterns at BC cell surface. These patterns rise as a resource to identify potential targets on TAM in the BC context, leading to the development of novel immunotherapies. Herein, we will summarize recent studies describing advances in identifying altered glycan structures in BC cells. We will focus on BC-specific glycosylation patterns known to modulate the phenotype and function of macrophages recruited to the tumour site, such as structures with sialylated or N-acetylgalactosamine epitopes. Moreover, the lectins present at the surface of macrophages reported to bind to such antigens, inducing tumour-prone TAM phenotypes, will also be highlighted. Finally, we will discuss and give our view on the potential and current challenges of targeting these glycan-lectin interactions to reshape the immunosuppressive landscape of BC.

## 1. Introduction

Breast cancer (BC) is one of the most prevalent cancer types, especially among women, with over 2 million new cases estimated for 2020 (24.5% from all new cases) [1]. Endocrine and targeted therapies have significantly improved the prognostic of hormone positive and human epidermal growth receptor 2 (HER2)-positive breast cancer, respectively, whereas for patients with triple negative BC (TNBC), scarce therapeutic options are available [2]. About 30% of patients with early-stage breast cancer have recurrent disease [3], as resistance to hormonal and targeted therapies is common and the broad range therapeutics (chemo or radiotherapy) show reduced effect in TNBC patients with advanced stages of the disease [2]. The lack of efficacy showed for patients is due to the heterogeneous nature of BC, which is currently described to be driven mainly by the surrounding microenvironment [4,5]. Thus, in recent years, there has been a shift in BC research from the tumour cells to the dynamic interactions of these cancer cells with their microenvironment. The growing appreciation of the tumour microenvironment (TME) as key determinant of BC progression shifted the focus of therapeutic discovery towards TME-targeted therapies, namely stromal-targeted and immunomodulatory therapies to revert immunosuppression [2,5].

Glycosylation is the most common post-translational modification of proteins, whereby glycans are attached to a protein for the synthesis of glycoprotein glycoconjugates. It is a highly regulated process that is affected by the physiological state of the cell or its pathological environment, such as in the oncogenesis process. Indeed, altered cell surface glycosylation is known as a distinct hallmark of cancer. The recognition of glycans that occur specifically in tumour cells has been described to modulate the immune microenvironment, playing a critical role in tumour cell biology and in the process of tumour immune escape and immunomodulation (reviewed in [6,7,8,9]). For instance, some immune checkpoint inhibitors, which have a key role in mediating the immunosuppressive response, are known to be highly glycosylated, inhibiting cytotoxic activity in infiltrated T-cells [6]. More recently, macrophages within the tumour site have also been reported to recognize tumour-specific glycans, which triggers their polarization into immunosuppressive phenotypes [9]. These tumour-associated macrophages (TAM) are described to suppress other immune cells and remodel the surrounding microenvironment, playing a key role in tumour progression (as detailed in Section 2) [10]. Major players in this glycan recognition are the glycan-binding receptors displayed at the immune cell surface, generally termed lectins. Lectins are transversal to different domains of life and are defined as proteins that recognize glycan structures in a highly specific, often multivalent, manner and that convey this recognition and structural information into functional cellular responses [11,12,13]. The biological functions of lectins are thus well recognized in a plethora of different cellular pathways. For instance, lectins are involved in immune cell function and adhesion and in pathogen recognition, either by recognizing endogenous mammalian glycans or binding to glycans on microorganisms [12,13].

Herein, we will review the current state on built-up of immunosuppressive microenvironments in BC, summarizing current knowledge on the role of glycan-mediated interactions between BC cells and immune cells, with a focus on TAM. We will address the altered cell-surface protein glycosylation present in BC and the main mechanisms described to induce such changes. We will review the impact of glycan-driven interactions on TAM modulation, focusing on key glycan epitopes and lectins reported to induce a protumour macrophage phenotype. Finally, we will discuss the potential and the current challenges to unravel these interactions as new targets for immunotherapies.

## 2. Tumour-Associated Macrophages within the Breast Cancer Microenvironment

**Figure 1:**
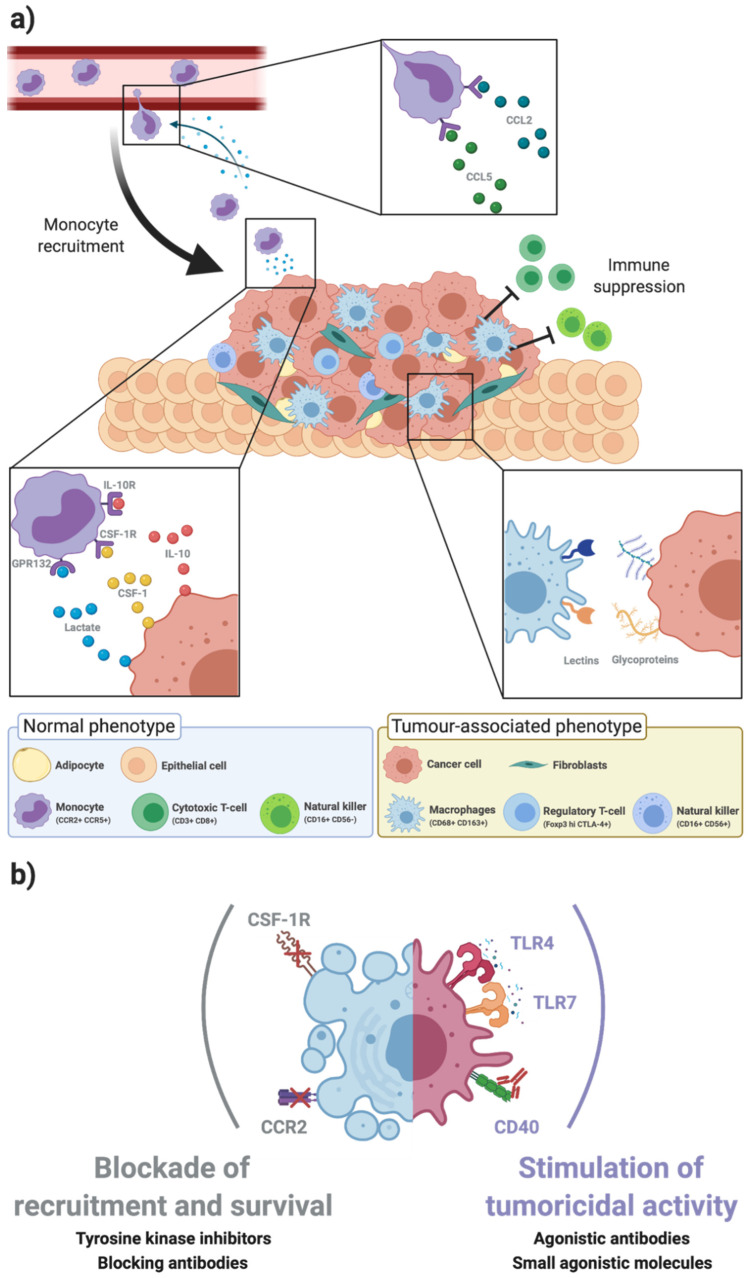
Tumour-associated macrophages modulate the breast cancer microenvironment. (**a**) The breast cancer (BC) microenvironment is composed by stromal cells (as fibroblasts, adipocytes, endothelial cells) and the immune infiltrate. Immune cells, such as T lymphocytes, natural killer (NK) cells and monocytes, are recruited to the tumour site at early stages. Monocytes are recruited from the bloodstream by chemokines secreted by the tumour cells, such as C-C motif chemokine ligand 2 (CCL2) and CCL5 (top inset). Within the BC microenvironment, monocytes polarize into immunosuppressive tumour associated macrophages (TAM), in a process regulated by cytokines, growth factors, and other signals secreted by tumour and stromal cells, such as interleukin 10 (IL-10), colony stimulating factor 1 (CSF-1), and lactate, recognized by the IL-10 receptor (IL-10R), the CSF-1 receptor (CSF1R), and G protein-coupled receptor 132 (Gpr123) (bottom left inset). The recognition of highly glycosylated proteins on tumour cells, such as mucin 1 (MUC1) and cluster of differentiation 24 (CD24), by immune glycan-binding receptors (lectins) also contribute to polarization into immunosuppressive TAM phenotypes (bottom right inset). TAM represent a high fraction of the microenvironment of human epidermal growth factor receptor 2 (HER2) overexpressing BC and triple negative BC (TNBC), being implicated in the modulation of stromal cells into a protumour phenotype and suppression of other immune cells, such as regulatory T cells and NK cells. (**b**) Macrophages as targets for novel immunotherapies. Due to the relevance of TAM in promoting an immunosuppressive BC microenvironment, they have been proposed as therapeutic targets for immunotherapy. Strategies that have reached clinicals trials include (i) blockade of the axes responsible for macrophage recruitment and survival, such as CCL2 and CSF-1 and (ii) stimulation of TAM tumoricidal activity, by triggering the TLR4, TLR7 or CD40-mediated pathways [4,21,22,23].

The BC microenvironment is composed mainly by stromal cells (fibroblasts, endothelial cells, adipocytes), immune cells, and the soluble factors and extracellular matrix (ECM) components they secrete (Figure 1a) [4,5]. The intricate molecular crosstalk between BC and the stromal components has been recently reviewed in [14], highlighting the therapeutical opportunities for stromal cells.

The immune infiltrate has been under the spotlight with the recent development of immunotherapies designed to trigger immune activation towards BC [15]. In BC, the immune fraction represents a relevant portion of the TME [4] and is mainly composed by lymphocytes, NK cells, and macrophages [16]. Recently, a deep single-cell proteomic atlas proposed by Wagner et al. showed that the immune landscape of BC patient samples was enriched in B and T cells, being the latter more abundant [17]. Noteworthy, this study along with others, depicted a high immune infiltration in the more aggressive BC subtypes, namely HER2 overexpressing BC and TNBC [2,16,17]. In fact, tumour infiltrating lymphocytes (TIL) are frequently found in highly proliferative tumours, being associated with favourable outcomes and nowadays TIL score prognostic and predictive value is well established, for TNBC and HER2-positive BC, as reviewed elsewhere [18,19]. Besides T cells, high prevalence of immunosuppressive macrophages with different phenotypes were also observed, along with minor populations of other immune cells [16,17,20].

Within the BC TME, BC cells are described to express ligands such as programmed death-ligand 1 (PD-L1) at their surface or increase the surface expression of receptors like cytotoxic T-lymphocyte-associated protein 4 (CTLA-4) in T cells [24]. These immune checkpoints sustain the binding of BC cells with immune cells, suppressing the inflammatory and tumoricidal functions of the latter [24]. In the light of the discovery of immune checkpoints, several breakthroughs have been achieved in recent decades [25,26]. The antibody-based immunomodulators targeting PD-L1 and CTLA-4 were the first generation of immune checkpoint inhibitors [15]. These strategies were developed to block the binding of cluster of differentiation (CD) 86 on antigen presenting cells (APC) and PD-L1 on tumour cells to the inhibitory receptors CTLA-4 and programmed death receptor-1 (PD-1) on T cells (respectively), sustaining the tumoricidal activity of cytotoxic effector T cells upon interaction with tumour cells [27].

So far, from several clinical trials for immune checkpoint inhibitors in BC patients, only Pembrolizumab, an anti-PD-L1 antibody has been approved, used in combination with chemotherapy [28]. Nevertheless, the response rate to the therapy only showed a positive outcome in a small subset of patients [28,29]. Several clinical trials were recently and are currently performed for targeting PD-L1, particularly in TNBC [24,30]. However, the responses to the therapies developed remained low, especially when used as single agents [30]. These studies provided insights of the need for a complementary strategy to improve the outcome of current immunotherapy, e.g., novel compounds for combination therapy. In addition, expanding the targets for immunotherapy to other immune cells may also reinforce the reshaping of the immune landscape and therefore improve patient outcome.

In the search for new approaches to expand immunomodulatory therapies, macrophages arise as targets of interest within the BC immune landscape [5,10,25]. This derives from the high macrophage infiltration observed in aggressive BC subtypes, particularly in TNBC, and the high correlation between macrophage infiltration and poor prognosis/clinical outcome [2,17]. TNBC is defined as a wide BC subtype, including all BC that do not express neither ER and progesterone receptor nor HER2. Hence, TNBC can be divided into six molecular subtypes: basal-like 1, basal-like 2, immunomodulatory, mesenchymal-like, mesenchymal-stem like, and luminal androgen receptor [20]. In an extensive analysis of a patient cohort, Jézéquel et al. defined two clusters of TNBC samples, which were functionally classified as involved in “Cell adhesion, locomotion and chemotaxis” (designated as C2) or “Immune response” (C3), according to gene ontology (GO) biological process terms [20]. Through analysis of gene expression signatures, the authors further observed that M2-protumorigenic macrophages were highly represented in the C2 cluster, associated with the basal-like BC subtype and TIL were enriched in C3, associated with the claudin-low subtype. This M2-enriched subtype showed a low immune response, indicating an immunosuppressive microenvironment, which was correlated with a poor outcome [20]. In fact, TAM regulate an immunosuppressive microenvironment in BC, by suppressing the functions of T cells, which decreases the efficacy of T cell immune checkpoint inhibitors [25,28]. TAM secrete immunosuppressive cues like IL-10, C-C motif chemokine ligand (CCL)18, CCL24 and transforming growth factor β (TGF-β), that polarize infiltrated immune cells into immunosuppressive phenotypes [29,30,31]. TAM also present PD-L1 at their surface, which binds to PD-1 of T-cells, inhibiting the cytotoxic function of the latter [32]. BC-stimulated TAM mediate tumour progression not only by suppressing other immune cells but also by remodelling the TME, allowing BC cell migration [21,25] and favouring neovascularization [22], facilitating the extravasation of BC cells into the blood stream and metastasis at distant sites [22,23,33].

The tumour-prone TAM phenotype arises from the crosstalk between monocytes recruited from the blood stream and BC cells (Figure 1a). BC cells secrete inflammatory factors such as CCL2 and CCL5, which recruit monocytes from the peripheral blood to the tumour site [10,31]. Once within the BC microenvironment, they are exposed to a hypoxic microenvironment, enriched in soluble factors like interleukin (IL)-10, IL-4, colony-stimulating factor 1 (CSF-1), TGF-β and lactic acid [10,21,22,23,32]. Prosurvival signalling from CCL2 and CSF-1 induces the phosphorylation of kinases from the Rho family and MAPK-ERK pathway (respectively), which mediates macrophage motility and proliferation [33,34]. In addition, other immunosuppressive cues trigger a response in macrophages, by activating signalling cascades such as signal transducer and activator of transcription (STAT)3 and STAT6 and hypoxia inducible factor (HIF)1α, which act on resolution of inflammation [35,36].

In the context of the complex molecular crosstalk within the BC microenvironment, a growing body of evidence is showing that polarisation of TAM also occurs via recognition of highly glycosylated membrane-bound proteins on tumour cells (e.g., MUC1 and CD24) by lectins on macrophages (Figure 1a). These specific interactions trigger an inhibitory signalling on macrophages suppressing the antitumour immune response and facilitating tumour invasion. The major tumour-associated glycan patterns in BC and their specific recognition by macrophage-expressed lectins will be the focus of Section 3 and Section 4, respectively.

## 3. Altered Glycosylation Patterns in Breast Cancer

**Figure 2:**
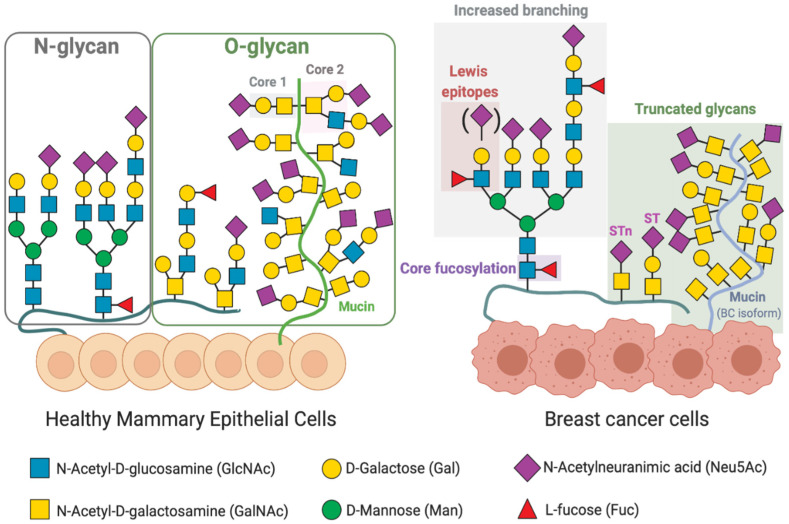
Altered glycosylation patterns of surface glycoproteins on breast cancer cells. Healthy mammary epithelial cells present at their surface diverse glycans, which are attached by a N-Acetylglucosamine (GlcNAc) to an asparagine (Asn) site—N-glycans—or by a N-Acetylgalactosamine (GalNAc) to a serine/threonine site (Ser/Thr; mucin-type O-glycans). Upon tumorigenesis, BC cells acquire a deregulated synthesis of glycans, which influences interactions between tumour cells and the surrounding microenvironment. Altered glycosylation in BC is characterized by an increase in Lewis antigens (Lewis^a^ and Lewis^x^; the sialylated Lewis^x^ and Lewis^a^) and an increase in α1-6-core fucosylation and branching of N-glycans. Additionally, truncated O-glycans are highly expressed in BC usually with terminal sialic acid. Monosaccharides are represented according to the symbol nomenclature for glycans (SNFG) [8,37,39,41].

In BC, similarly to other cancer types, there is a deregulation in glycan biosynthesis that leads to changes in glycan patterns of cell surface glycoproteins, not observed in the healthy breast epithelial cells [7,37]. The most common cancer-associated glycosylation changes include a truncated O-glycan phenotype, increased branching of N-glycans and higher levels of fucosylation and sialylation of N- and O-glycans and (Figure 2).

The alterations in glycosylation are dependent on several intrinsic and cell-specific factors. Two mechanisms have been initially proposed by Hakomori and Kannagi for the altered glycosylation in cancer: (i) incomplete synthesis of glycans and (ii) neosynthesis of glycans [38]. Incomplete synthesis encompasses an impairment in the synthesis of the correct structure of glycans, commonly by the overexpression of glycosyltransferases leading to truncated glycan structures [7,37]. In neosynthesis, there is a cancer-induced increased synthesis of glycan structures, usually present in healthy breast tissues (overexpression of antigens), or the synthesis of novel structures (neoantigens), unique to BC cells [39,40]. These abnormal glycosylation patterns derive mainly from changes in the expression level of glycosyltransferases and their localisation in the Golgi, as well as differences in the availability or abundance of sugar nucleotide donors and cofactors [36]. Incomplete synthesis is usually observed in early-stage cancer, in opposition to neosynthesis, which is more common in late-stage or more aggressive BC subtypes [7,39].

The most common truncated glycan structures observed in BC are on O-glycans of heavily glycosylated mucin or mucin-type O-linked glycoproteins. Mucin type O-glycosylation (also referred to as O-GalNAc glycosylation) is characterised by the addition of GalNAc to serine and threonine residues of the peptide backbone, which is mediated by a large family of polypeptide N-acetylgalactosaminyltransferases (GalNAcTs). In normal breast epithelial cells, the addition of other monosaccharides occurs, e.g., galactose to form the core 1, which is converted into core 2 by the addition of GlcNAc to form the branched core 2 structure. O-glycans expressed by normal breast epithelial cells are mainly composed of core 2 structures extended with a linear polylactosamine chain, which can be fucosylated or sialylated (Figure 2) [42,43,44]. In contrast, breast cancer cells express glycoproteins with truncated O-glycan antigens, including: the Tn (GalNAcα-Ser/Thr) and T or Thomsen–Friedenreich (Galβ1-3GalNAcα-Ser/Thr) antigens, and their sialylated counterparts, sialyl-Tn (STn; Neu5Acα2-6GalNAcα-Ser/Thr) and sialyl-T (ST; Neu5Acα2-3Galβ1-3GalNAc) antigens [7]. As examples, the ST- or STn-enriched MUC1 mucin glycoforms are commonly overexpressed in BC cells, when compared to normal mammary tissue [45]. MUC1 has been reported to influence the epidermal growth factor receptor (EGFR) signalling in BC. The authors described that the MUC1 glycoform with truncated glycans stabilised EGFR signalling in a Luminal BC cell line, by protecting the receptor from ubiquitination [46]. Additionally, ST-enriched MUC1 was also described to modulate the BC immune microenvironment [47], as discussed below (Section 4).

The changes in O-linked glycans described are mainly due to changes in the expression of GalNAcTs and sialyltransferases, which hamper extension of the glycan chain and originate truncated structures (Figure 2) [7]. There are twenty GalNAcTs able of transfer GalNAc to Ser/Thr, with different peptide substrate specificity [48]. The expression of specific GalNAcTs is tissue-dependent, being GalNAcT 1 and 2 commonly found in healthy mammary tissues [7]. In BC, however, increased expression of GalNAcT 6 [49] and GalNAcT 14 [50] has been reported. Park et al. observed increased BC cell adhesion upon silencing of GalNAcT6 or MUC1, suggesting that overexpression of GalNAc might contribute to mammary carcinogenesis through aberrant glycosylation and stabilization of MUC1 [51].

ST6GalNAc I converts Tn into STn, inhibiting the extension upon GalNAc residues. ST3GalI and ST6GalNAcII also convert T antigens into ST, competing therefore with C2GnT1 and inhibiting the formation of core 2 glycans (Figure 2) [7]. Differences in the expression levels of glycosyltransferases involved in O-glycosylation have been observed between Oestrogen receptor (ER) positive BC and TNBC, both in patient material and in cell lines [7]. The data suggests that ER+ carries mainly short core 1 glycans in O-linked glycoproteins whereas TNBC can have an increase in core 2 structures [7,39] (Figure 2).

Another glycosylation alteration frequently associated with a cancer phenotype is the increased expression of complex β1,6-branched N-linked glycans [52,53]. This altered glycosylation is a result of the increased activity of N-acetylglucosaminyltransferase V (GnT-V), encoded by the mannoside acetylglucosaminyltransferase 5 (MGAT5) gene, which is upregulated in cancer [53]. Branched N-glycans are further elongated with complex poly-N-acetyllactosamine chains (poly-LacNAc, repeats of Galβ1,4GlcNAcβ1,3-) capped with sialic acid and fucose (Figure 2). GnT-V was found to regulate early events in breast carcinoma development using a HER2-transgenic mouse mammary tumour model [54]. In addition, downregulation of GnT-V in mouse mammary cancer cell lines resulted in a significant suppression of tumour growth and metastasis [54]. Recently, increased poly-LacNAc structures have been associated with advanced HER2+ and TNBC tissues [55].

These and other studies in patient cohorts described BC-specific aberrant N-glycosylation signatures in the serum and tissue of patients. De Leoz et al. characterized the N-glycosylation patterns of glycoproteins in patient’s serum by matrix-assisted laser desorption/ionization (MALDI)-Mass spectrometry (MS); they reported an increase in high mannose structures in BC patients compared to healthy individuals, suggesting that it might be a feature of tumour progression [56]. Indeed, it has been reported that high mannose forms of EGFR expressed on the cell surface of several lung cancer cell lines were specifically recognized by an endogenous lectin, surfactant protein D, leading to downregulation of EGF signalling and consequently tumour-suppressive effects [57]. A high mannose form of EGFR has also been identified as the target of a therapeutic anti-EGFR antibody (A806) in cell lines from distinct tumour types overexpressing the receptor [58]. Lomax-Browne et al. analysed by MALDI-MS the glycans on immunoglobulin A1 (IgA1) from the serum of patients with metastatic and nonmetastatic BC, in comparison with healthy donors. The authors reported an increase in T antigen and sialylation, in O- and N-glycans respectively, in serum IgA1 of BC patients. Lectin binding assays confirmed increased sialylation (α2,6-linked sialic acid) [59].

Recent studies took advantage of MALDI imaging MS to characterize the specific distribution of glycans within the cancer tissue. [55,60,61]. The authors reported the detection of high-mannose, branched (tri- and tetraantennary structures) and fucosylated glycans within tumour regions and unveiled regional specificities within the tumour masses (e.g., necrotic regions enriched in N-glycans with limited branching, containing sialic acid modifications and lacking fucose modifications) [55,60]. Lee et al. reported elevated levels of highly sialylated and fucosylated complex type N-glycans in five BC cell lines relative to normal mammary epithelial cells. The authors observed differences in fucosylation and sialylation between the cell lines representing different BC subtypes. The diversity of analytical techniques employed so far to profile the N-glycosylation of BC tissue and BC cell lines, as well as the restricted number of samples analysed in each study, limit the conclusions that can be drawn regarding BC subtype-specificity and the correlation between cell lines and tumour tissue.

Fucose residues are present in N-glycans as core fucose (core fucosylation) and in N-glycans and O-glycans as terminal epitopes (terminal fucosylation), comprising Lewis blood-group antigens [62]. Core fucosylation (α1-6-linked fucose) consists in the addition of α1-6-fucose to the innermost GlcNAc residue of N-glycans through the action of fucosyltransferase 8 (encoded by FUT8) [63] and has been described in key BC receptors. For example, increased core α1-6-fucosylation of EGFR was associated with increased dimerization and phosphorylation, which resulted in increased EGFR-mediated signalling associated with tumour cell growth and malignancy [64]. Tu et al. also showed that core α1-6-fucosylation of TGF-β receptor facilitated the dimerization of the receptor and promoted higher activation of SMAD2/3, consequently inducing higher epithelial to mesenchymal transition (EMT) in BC cell lines embedded in an ECM (the basement membrane mimetics, Matrigel) [65].

The Lewis blood group antigens have been described as common terminal epitopes of BC N-glycans and O-glycans, mediating the binding of BC cells to the endothelium [40,63]. Membrane-bound glycoproteins rich in sialyl Lewis X (SLex; Neu5Acα2-3Galβ1-4[Fucα1-3]GlcNAcβ-R) and sialyl Lewis A (SLea; Neu5Acα2-3Galβ1-3[Fucα1-4]GlcNAcβ-R), such as mucins or CD44, are known ligands for P- and E-selectin at the surface of endothelial cells, mediating the extravasation of BC cells into the blood stream and seeding at distant sites [39]. SLex and Lewis x (Lex; Galβ1-4[Fucα1-3]GlcNAcβ-R) have been reported to localise specifically to the invasive front of BC specimens, by immunohistochemical analysis [66]. The relevance of Lex and SLex as modulators of angiogenesis and metastasis of BC cells and in BC drug resistance has been recently reviewed elsewhere [67].

## 4. Tumour-Associated Macrophages Recognise Altered Glycosylation Patterns on Breast Cancer Cells

**Figure 3:**
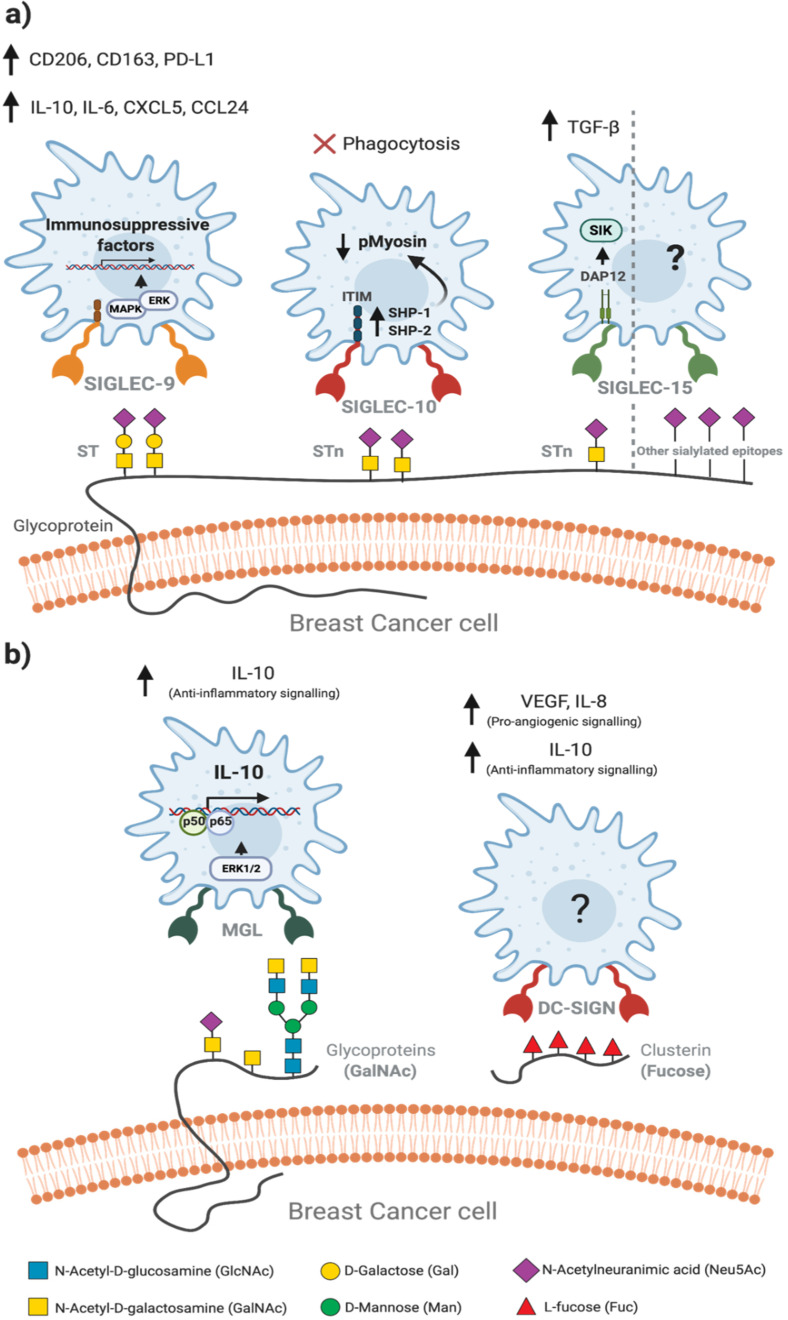
Glycan-lectin interactions within the breast cancer microenvironment modulate the phenotype of tumour-associated macrophages. The binding of lectins (glycan binding receptors) on macrophages to aberrant glycans exposed at the surface of BC cells induces a protumour phenotype in macrophages, promoting immunosuppressive functions. (**a**) Few glycan-lectin interactions are currently known, being the most described the interactions between sialylated epitopes of BC cells and sialic acid-binding immunoglobulin-like lectins (SIGLECs). The recognition of ST-enriched MUC1 by SIGLEC-9 activates the MAPK-ERK pathway, increasing the expression of immunosuppressive factors, which induces the protumour phenotype of other infiltrating immune cells. Sialyl T (ST) and sialyl Tn (STn) antigens are recognized by SIGLEC-10, triggering an immunoreceptor tyrosine-based inhibitory motif (ITIM)-mediated signalling which recruits Src homology 2 (SH2)-domain-containing protein tyrosine phosphatase 1 (SHP1) and SHP2 tyrosine phosphatases. These phosphatases inhibit the phosphorylation of myosin, hampering the motility of macrophages membrane and engulfment of tumour cells. SIGLEC-15 has been described to bind STn, inducing a DAP12-mediated cascade that triggers an increased secretion of TGF-β. In another recent study, SIGLEC-15 showed higher binding avidity for other sialylated glycans, not triggering TGF-β secretion, but instead activation of the SYK/MAPK signalling pathway. SIGLEC-15-glycan interactions within TME remains an open question. (**b**) Terminal GalNAc on tumour cells is recognized by macrophage galactose-like lectin (MGL), upregulating the ERK pathway and the expression of IL-10, via NF-κB. Some studies also report the role of clusterins in TAM modulation. Although much is not understood in this interaction, clusterin-stimulated macrophages were shown to polarize into a proangiogenic, anti-inflammatory phenotype, with increased secretion of VEGF, IL-8 and IL-10. Monosaccharides are represented according to the symbol nomenclature for glycans (SNFG) [9,41,47,61,68,69,70,71,72,73,74].

There is an increased recognition of the role of aberrant cancer-associated glycosylation patterns on the immune regulation of the TME. Tumour-associated glycans are recognized by lectin receptors expressed on the plasma membrane of immune cells, eliciting immunogenic or immunosuppressive pathways. Human macrophages express at their surface different lectin receptors, which include members of two structurally distinct families: the C-type lectins and the sialic acid-binding immunoglobulin-like lectins (SIGLECs), as reviewed by Brown & Crocker [12,68]. Herein, we will focus on lectins of these families that have been described to contribute to the immunosuppressive TAM phenotype. These include: SIGLEC-9, -10 and -15 (Figure 3a) and the C-type lectins, macrophage galactose-type lectin (MGL) and dendritic cell-specific ICAM-3-grabbing nonintegrin (DC-SIGN) (Figure 3b) [13,68,69].

SIGLECs typically transduce the signal via immunoreceptor tyrosine-based inhibition motif (ITIM)-mediated signalling cascades. Phosphorylated ITIMs recruit the SHP1 and SHP2 tyrosine phosphatases, which upon activation modulate signalling which ultimately lead to the polarisation towards immunosuppressive TAM phenotype (Figure 3a) [69]. In fact, sialic acids are essential determinants of self-recognition by the innate immune system including macrophages, acting as surface self-associated molecular patterns (SAMPs) and mediating the inhibition of inflammatory signalling cascades [70].

MUC1-ST, a BC glycoform (see Section 3), was specifically bound by SIGLEC-9 in BC infiltrating monocytes and macrophages, inducing a calcium influx associated with activating signalling, in contrast with the classical engagement with recruitment and activation of the phosphatases SHP-1 or SHP-2. The calcium influx led to the activation of mitogen-activated protein kinase (MAPK)–ERK pathway, inducing a protumour TAM phenotype [47]. The binding of SIGLEC-9 from human purified macrophages to recombinant MUC1-ST caused an increase in the secretion of IL-10 and IL-6, as well as in the expression of the surface marker CD163 [47] Recently, Beatson et al. also showed that the binding of MUC1-ST to SIGLEC-9 further increased the surface expression of CD206 and PD-L1, corroborating the immunosuppressive phenotype of MUC1-ST-induced macrophages [71]. Additionally, macrophages treated with MUC1-ST showed an increase in the expression of CXCL5, CCL24 and other anti-inflammatory cues, promoting the polarization of immunosuppressive neutrophils, which will further support the progression of the BC microenvironment [71].

CD24 is a highly sialylated glycoprotein, expressed in several BC subtypes, particularly in TNBC, which is correlated with poor patient survival [72]. CD24 is specifically recognised by SIGLEC-10 on macrophages, eliciting an inhibitory cascade mediated by the Src homology region 2 (SH2) domain, containing phosphatases SHP-1 and SHP-2. These phosphatases are associated with two ITIMs in the cytoplasmic tail of SIGLEC-10, which block the cytoskeletal rearrangement required for cellular engulfment by macrophages [72]. The blockade of CD24 in MCF-7 cells has been shown to promote higher phagocytosis by human macrophages, both in monolayer cultures of MCF-7 cells, cocultures with human macrophages, preconditioned with IL-4, IL-10, or TGF-β and in murine models [72].

Some recent studies have highlighted a role of SIGLEC-15, which is upregulated on TAM, in modulating the TME and favouring tumour suppression [73,74]. One proposed mechanism was through the engagement of the macrophage expressed SIGLEC-15 with tumour cells expressing the truncated STn antigen, which triggered the production of immunosuppressive TGF-β [73]. However, in another study the binding of SIGLEC-15 to sialylated glycans on human BC cell lines was shown to be independent of STn [75]. Indeed, glycan microarray analysis with structurally defined glycans showed that SIGLEC-15 binds with higher avidity to sialylated glycans other than STn or related antigen sequences. In addition, no enhancement of TGF-β secretion was observed following co-culture of SIGLEC-15-expressing monocytic THP-1 cell lines with tumour cells expressing STn or following SIGLEC-15 cross-linking with monoclonal antibodies. However, activation of the SYK/MAPK signalling pathway was observed, which may modulate the functional activity of macrophages [75]. The sialylated ligands recognised by TAM-SIGLEC-15 on tumour cells and the effects of their specific recognition in modulating the TME remain open questions.

In addition to the sialylated antigens, epitopes with terminal GalNAc are also recognised by macrophages through the MGL. MGL specificity for GalNAc residues with exposed C3- and C4-hydroxyl groups, explains the ability of the receptor to bind to truncated Tn and STn O-glycan antigens on tumour-associated isoforms of MUC1 and not to complex and branched O-glycan structures found on normal cells (Figure 2) [7]. Upon binding to GalNAc, MGL induces the phosphorylation of extracellular signal-regulated kinase 1,2 (ERK1,2) and nuclear factor-κB (NF-κB) activation, resulting in secretion of IL-10 associated with a protumorigenic TAM phenotype (Figure 3b) [9,76]. Other abnormal glycans expressed by BC cells have been reported to modulate macrophages [37,46]. Merlotti et al. reported an increase in fucosylated clusterins with terminal sialylation in BC tissues [77]. Clusterins are highly glycosylated glycoproteins, being one of the most prominent extracellular chaperones, involved in scavenging and clearance events [78]. Fucosylated clusterins bind to DC-SIGN present at the surface of macrophages, inducing a proangiogenic phenotype with high secretion of vascular endothelial growth factor (VEGF), IL-8 and IL-10 [77]. Notably, macrophages treated with fucosylated clusterins showed decreased expression of HLA-DR and CD86, typical of inflammatory macrophages (Figure 3b) [77].

## 5. Glycan-Lectin Interactions: Novel Targets for Tumour-Associated Macrophage-Based Immunotherapy in Breast Cancer?

Given the diversity of potential targets presented by TAM for the development of novel immunotherapies to tackle BC, several trials have already been pursued to block the axes that induce monocyte recruitment and mediate TAM functions [31,79,80]. In fact, Pathria et al. recently reviewed the status of therapeutics targeting key TAM-related axes, such as CCL2-CCR2, CSF-1-CSF-1R (Figure 1a), currently in Phase I/II of clinical trials. The authors also reviewed other approaches designed to target TAM, which comprised the stimulation of Toll-like receptor (TLR)-4, -7 and -9, or CD40, to restimulate the tumoricidal effects in TAM (Figure 1b). Despite promising, the preclinical results of TAM-targeting immunotherapies into the clinics have been showing limited translation.

With a growing body of evidence pointing out the relevance of glycosylation in the modulation of macrophages [9,69,81], targeting glycans in key ligands and receptors has been proposed as potential for novel therapies to tackle BC [9,81,82]. Bertozzi and coworkers explored a related approach to target NK cell immunosuppression in HER2-overexpressing BC [83]. The authors developed an anti-HER2 antibody–sialidase conjugate that was shown to bind to HER2, blocking its downstream signalling in BC cells and removing sialic acid specifically from HER2+ BC cells. In antibody-dependent cell-mediated cytotoxicity assays with BC cell lines and NK cells, the authors showed that the sialidase activity blocked the binding of HER2+ tumour cells to NK cells via SIGLECs, therefore improving the antibody-dependent cellular cytotoxicity (ADCC) response [83].

Direct interference on BC glycan-TAM lectin interactions or on glycoepitope synthesis/presentation by tumour cells are two potential approaches for immune modulation of the TME that remain largely unexplored. Beatson et al. used primary tumour and immune cells, both extracted from patient samples, showing the importance of the interaction between SIGLEC-9 and mucin-derived truncated antigens for the immunosuppressive phenotype of TAM, as described in Section 4 [47,71]. In another study, Gray et al. employed syngeneic BC models to assess the potential of targeting glycan degradation to improve anticancer immune response [84]. Sialidase-targeted BC cells induced an increase in total tumour leukocytes, with a reduced number of CD206+ macrophages. These studies support the possibility of switching macrophage phenotype by targeting immunosuppressive glycan-lectin interactions. The authors showed that the recognition of sialic acid by macrophages was mediated by murine SIGLEC-E [84], a functional orthologue of human Siglec-9. Thus, the clinical translation of these findings will be dependent on their confirmation in a human setting. These examples highlight the importance of human-relevant experimental models to improve the translational potential of the research.

Many of the preclinical studies reported so far on targeting TAM employed: (i) nonhuman models, which may vary in tumour cell glycosylation patterns, stromal and immune cell receptors and in receptor-ligand affinities, as it occurs for cytokines in murine models [85]; (ii) cell models usually relying on two-dimensional (2D) monolayer cultures of tumour cell lines that do not represent the BC TME components nor the three-dimensional (3D) architecture of their complex interactions [86,87].

Recently, efforts in the development of animal models for immuno-oncology have been focused on humanized mouse models, as reviewed in [85,88]. Human immune interactions within the TME can be investigated allowing, for instance, the screening of human-specific cancer immunotherapeutic drug candidates [88]. Engraftment of human hematopoietic stem cells results in the development of autologous human immune system, with matching human leukocytic antigens (HLA) [88]. Nonetheless, these approaches still show limitations, such as only partial HLA-matching or the presence of mouse cytokines that do not cross-react with human receptors, leading to the suboptimal development of specific human immune cell types in humanized mice, such as macrophages [85,88].

Extensive efforts are also being made towards the development of in vitro and ex vivo human model systems that can represent the complexity and dynamics of BC microenvironment, taking advantage of bioreactor, microfluidics, bioprinting and other advanced cell culture technologies [89,90,91,92]. We have recently developed a 3D coculture model of the TME, that includes tumour cells, fibroblasts, and immune cells [93,94]. We reported crosstalk between the different cell compartments that result in the build-up of immunosuppressive microenvironments, with polarization of TAM with a tumour-supporting phenotype [94,95]. Importantly, the impact of the cell culture system on the glycosylation of tumour cells has started to be recognized. David and coworkers reported that 3D cultures of ovarian cancer cell lines showed a higher resemblance in terms of mucin and truncated O-glycan footprint to tumour tissue than the same cell lines cultured as 2D cultures [96]. Other reports followed in gastric and breast cancer [97,98]. For the latter, the authors describe different N-glycosylation patterns for the same BC cells, when cultured in 2D, 3D and as mouse xenografts [98].

Another important aspect is that most studies were target-oriented, following up on limited subset of glycans described in pathogen recognition [82,99]. Moreover, given the redundancy and promiscuity of TAM ligands and receptors and the compensatory signals from the TME, such as secondary glycan-lectin interactions or cytokine-receptor interactions [9,82,100], it can be anticipated that simplistic single blockage of specific glycan-lectin or cytokine-receptor interactions will not be enough to inhibit or to revert immunosuppressive TAM. Untargeted profiling of glycan patterns at the surface of BC cells recognized by lectins on TAM will be required to achieve a comprehensive characterization of the glycan-lectin interactions that drive the TAM immunosuppressive phenotype within the BC microenvironment. The intrinsic heterogeneity characteristic of BC, as well as potential specificities in the glycosylation pattern of BC subtypes demand further efforts in glycosylation profiling. This becomes even more complex for TNBC, which encompasses six distinct molecular subtypes, as described in Section 2, and presents the highest diversity among patients [20]. The recent developments in MS for glycomics and glycoproteomics and in mass spectrometry imaging allowing to uncover molecular information of glycosylation changes should prove great technological advances to the definition of the BC glycomes [101,102]. Other emergent tools in glycomics, such as glycan microarrays offer powerful high-throughput and high-sensitive tools for studying glycan-lectin interactions [103] and for glycan ligand discovery from glycomes [104,105]. These tools are expected to overcome the main limitations of less miniaturized or targeted approaches. In combination with these technologies, the continuous development of structural biology methods, including innovative paramagnetic-based nuclear magnetic resonance (NMR) methodologies [106], will add molecular detail and structural information to expedite structure-function studies.

In the era of immunomodulatory therapies, the recent advances in functional glycomics, structural glycosciences, and in cancer model systems are paving the way towards the clinical application of immunotherapies targeting TAM via glycan-lectin interactions.
